# Is Biologic Therapy an Effective Tool for Achieving Remission in Severe Asthma? A Retrospective Study in Central Romania

**DOI:** 10.3390/life15071113

**Published:** 2025-07-16

**Authors:** Corina Mărginean, Dragoș Huțanu, Mara Andreea Vultur, Hédi-Katalin Sárközi, Edith-Simona Ianoși, Maria Beatrice Ianoși, Andreea Safta, Gabriela Jimborean, Corina Eugenia Budin

**Affiliations:** 1 Oncology and Palliative Care Department, George Emil Palade University of Medicine, Pharmacy, Science and Technology of Târgu Mureș, 540139 Târgu Mureș, Romania; corina.marginean@umfst.ro; 2 Pulmonology Department, George Emil Palade University of Medicine, Pharmacy, Science and Technology of Târgu Mureș, 540139 Târgu Mureș, Romania; mara.vultur@umfst.ro (M.A.V.); hedi.balog@umfst.ro (H.-K.S.); edith.ianosi@umfst.ro (E.-S.I.); gabriela.jimborean@umfst.ro (G.J.); 3 Pulmonology Clinic, Mureș County Clinical Hospital, Târgu Mureș, 540011 Târgu Mureș, Romania; ianosi.maria-beatrice@stud18.umfst.ro (M.B.I.); andreeasafta260297@gmail.com (A.S.); 4 Pathophysiology Department, George Emil Palade University of Medicine, Pharmacy, Science and Technology of Târgu Mureș, 540139 Târgu Mureș, Romania; corina.budin@umfst.ro

**Keywords:** severe asthma, biologic therapy, asthma remission

## Abstract

Background: Severe asthma, which is characterized by persistent symptoms despite standard therapies, presents a significant clinical challenge. Biologic therapies targeting specific inflammatory pathways offer a potential avenue for achieving disease remission. This retrospective study evaluates the effectiveness of biologic therapies in achieving remission in severe asthma within a central Romanian cohort. Methods: Forty-eight patients with severe asthma treated with omalizumab, benralizumab, or dupilumab (2020–2025) were assessed. Clinical remission was defined using ACT scores, exacerbation frequency, corticosteroid use, and FEV1. Biological remission was determined using FeNO and eosinophil levels. Statistical analysis was performed to compare treatment outcomes. Results: At 12 months, 75% of patients achieved biological remission, and 75% reached clinical remission criteria. Significant improvements were observed in FEV1 (*p* < 0.001), eosinophil counts (*p* < 0.001), and ACT scores (*p* < 0.001). Complete remission, encompassing clinical, biological, and functional normalization, was observed in 54.2% of patients. Conclusion: Biologic therapies demonstrate promise in inducing comprehensive remission in severe asthma, supporting their role in improving disease control and lung function. Further research with larger cohorts is warranted.

## 1. Introduction

Asthma, which is a heterogeneous disease categorized as obstructive lung disease [1], is currently one of the non-communicable diseases causing significant morbidity worldwide, especially in urban areas, due to its increasing prevalence. As stated by Lommatzsch et al. in 2022, the underlying pathophysiology of asthma involves various complex inflammatory pathways, leading to the development of monoclonal antibodies targeting effector molecules of the inflammatory cascade [2]. The use of these innovative therapeutic classes has led to improved disease control and even remission [3].

The concept of remission in asthma is a controversial notion whose criteria are still debatable. Complete remission, initially developed in rheumatoid arthritis or oncology, was later considered for inflammatory bowel disease [2]. If, for rheumatoid arthritis, the concept of remission refers to the minimum dose of medication to keep inflammation at its lowest level, for asthma, assumptions have been difficult to establish. In defining the concept of clinical remission, the most recent criteria include the absence of symptoms as assessed using the asthma control test (ACT) or asthma control questionnaire (ACQ), the absence of exacerbations, and the elimination of the use of systemic corticosteroids. These three criteria can be added to a functional criterion that refers to lung function and implies a decline in forced expiratory volume in 1 s (FEV1) of <5% or a predicted FEV of ≥80%. A fractional exhaled nitric oxide (FeNo) level of <25 ppb and a blood eosinophil level of less than 300 elements per microliter are parameters to be considered when biological remission is the target. The sum of the criteria required for clinical remission and biological remission results in complete remission [4].

Different levels of remission are associated with the use of different classes of biologic drugs in the management and treatment of severe asthma, and the parameters used may vary depending on the specific situation [2,3]. The goal of asthma control as a single goal was replaced by the concept of clinical remission in 2023, leading to a paradigm shift in asthma management. Initially, the concept of clinical remission referred only to a period lacking symptoms, but later on, it implied achieving the above-mentioned criteria with maintenance of treatment (especially biologic therapy), which led various National Respiratory Societies to include the concept of clinical remission in national guidelines [5].

Identifying the clinical phenotype and inflammatory phenotype is essential for achieving the remission threshold in asthma [6]. The inflammatory processes underlying the clinical phenotypes of asthma are categorized into two primary global inflammatory phenotypes that are determined by the predominant immunologic pathways that drive disease pathology: eosinophilic and non-eosinophilic [7,8]. Biomarkers (IL-4, IL-5, IL-13, IgE) function in different ways, but they ultimately converge and lead to an increase in inflammation. Hypereosinophilia is induced by increased IL-5 production, and B cells are mediated by IL-4, leading to IgE synthesis [9,10]. Nitric oxide production and smooth muscle contraction are regulated by IL-13, leading to bronchial hyperresponsiveness [11].

## 2. Methods

The objective of this study was to determine whether clinical, biological, or complete remission was achieved in patients with severe asthma 1 year after the initiation of biologic therapy (omalizumab, benralizumab, or dupilumab).

We aim to evaluate the clinical and biological remission criteria across different biological agents based on real-world data collected in Romania.

The eligibility for biologic therapy in severe asthma patients was determined by meeting established clinical and laboratory criteria, such as a documented history of frequent exacerbations despite high-dose inhaled corticosteroids and additional controller medications. Severity was defined according to the ERS/ATS severe asthma guidelines, with particular attention to eosinophil thresholds (≥150 cells/μL), the FeNo value, and Ig E measurements. The frequency of exacerbations (at least two exacerbations requiring systemic corticosteroids in the past year) was also taken into account. These criteria align with the local reimbursement rules.

The study included a total of 48 patients in the Pulmonology Clinic of the Mures County Clinical Hospital between 2020 and 2025 and was approved by the Ethics Commission with the number 937/23.01.2025. The patients included received treatment with benralizumab, omalizumab, or dupilumab.

All patients included in the study had a sustained diagnosis of severe asthma, which involves inhaler treatment consistent with the GINA guidelines [1] and national protocols. Prior to the initiation of biologic therapy, all patients were treated according to the GINA step 5 guidelines, receiving high-dose inhaled corticosteroids (ICSs) in combination with long-acting beta-agonists (LABAs). In addition, many patients were on other controller medications, such as leukotriene receptor antagonists (LTRAs), long-acting muscarinic antagonists (LAMAs), or theophylline. A considerable proportion also required intermittent or chronic use of oral corticosteroids (OCSs) to achieve partial symptom control.

In chronological order, biological medication in severe asthma was available in Romania as follows: omalizumab since 2008 by the Order of the Minister of Health [12] and the President of the National Health Insurance House, no. 1301/500/2008; benralizumab since 2021; dupilumab since 2022.

### 2.1. Inclusion Criteria

- A confirmed diagnosis of severe asthma according to the GINA guidelines and national reimbursement criteria in Romania.
- Eligibility for biologic treatment based on predefined thresholds, including ≥2 exacerbations/year despite high-dose ICSs/LABAs, a blood eosinophil count of ≥300/μL (for anti-IL-5 agents), or elevated total IgE levels with proven allergy (for omalizumab).
- Patients who had completed at least one year of biologic therapy.
- Patients who had at least one follow-up at 1 year (ACT questionnaire, exacerbations, biomarkers, biomarkers, and respiratory function tests).

### 2.2. Exclusion Criteria

- Patients with a diagnosis of severe asthma but without criteria for inclusion in biologic treatment.
- Patients who had not completed 1 year of treatment at the time of assessment or for whom data were not available to assess remission.

### 2.3. Clinical Remission Was Assessed According to the Following

   I. ACT questionnaire analysis (at baseline, 3 and 6 months, and 1 year). An ACT score above 20 points was considered clinical remission.
  II. Assessment of exacerbations during the first 12 months of treatment. The absence of exacerbations during the 12-month follow-up period of treatment was considered remission.
III. The use of systemic corticosteroids (either chronic or intermittent treatment; for those with intermittent treatment, such as short courses of systemic corticosteroids, the cumulative dose per 12 months was calculated).
IV. Functional assessment by analyzing FEV1 values at baseline (T0), at 3 and 6 months, and at 12 months after initiation (T3). Clinical remission was considered at FEV1 values above 80%.

Patients with severe asthma included in the study were phenotyped prior to the administration of biological medication. The three biomarkers analyzed were total Ig E and specific Ig E, FeNo, and eosinophil count. After analyzing the results of these biomarkers, a therapeutic algorithm was applied. Thus, eosinophilia was a key criterion in the selection of anti-IL-5/IL-5R therapies such as benralizumab. Patients with higher blood eosinophils and frequent exacerbations despite standard treatment were typically referred to benralizumab, while those with signs of allergic sensitization and elevated IgE were considered for omalizumab. Patients who did not show the dominant phenotype (mediated by eosinophilic or IgE) were considered eligible for dupilumab treatment. At the time of inclusion of these patients in the study, the indication for treatment with dupilumab for chronic rhinosinusitis with nasal polyposis was not described. Among patients with an indication of benralizumab, those with nasal polyposis were considered super-responders [13,14], which is why our benralizumab group had the highest eosinophilia and the highest frequency of concomitant sinusitis.

The FeNo value (ppm) and serum eosinophilia value were used to assess biological remission. For FeNo, a cutoff of 20 ppb was used according to GINA 2021 [15]. Although the criteria published by Lommatzsch, Marek et al. in 2024 [5] established a cutoff for the value of eosinophils of 300 elements/microliter, in our study, we used 150 elements/microliter as a cutoff value for achieving biological remission. These two biomarkers have proven their usefulness in the literature for all three types of treatment analyzed in this study. For patients under treatment with omalizumab, it should be noted that the IgE value cannot be considered as a way to assess treatment response. For these patients, clinical remission assessment was specifically used. Serum eosinophils in patients with severe IgE-mediated asthma were followed in dynamics, appreciating a downward trend as a favorable outcome factor.

The evaluation of complete remission was assessed by summarizing the results of clinical and biological evaluations.

The analyzed parameters were differentially reported, and correlations were made while considering age, gender, and comorbidities (obesity, associated cardiovascular disease, and gastroesophageal reflux disease (GERD)), as well as individualized asthma treatment.

### 2.4. Statistical Analysis

The statistical analysis was performed with IBM SPSS Statistics version 26.0.0, where the distribution of quantitative data was tested using histograms, Q-Q plots, and, finally, the Shapiro–Wilk test for normality, confirming the presence of non-parametric data. Therefore, all quantitative data were expressed as the median (Q25–Q75). Qualitative data were analyzed using frequencies, with results expressed as *n* (%). Differences between study groups were analyzed using the Mann–Whitney test or Kruskal–Wallis test for independent samples, the Wilcoxon test or the Friedman test for related samples, and the Chi-Square test, setting the significance limit to α = 0.05.

## 3. Results

A total of 48 patients with severe asthma were included in the study. The median age was 67 years (range: 55–72), and 60.4% (29/48) were female. Most patients (72.9%) resided in urban areas. Among the biologic therapy groups, those receiving benralizumab were slightly older (median age: 68) than those taking dupilumab (65) and omalizumab (52), although the difference did not reach statistical significance (*p* = 0.08). Baseline demographic parameters, including gender distribution, BMI, asthma duration, age of onset, and smoking history, were not significantly different between the groups (Table 1).

Analysis of ear, nose, and throat (ENT) comorbidities demonstrated a comparable prevalence between groups. Allergic rhinitis affected approximately 28–29% of patients in each treatment cohort (*p* = 0.99). Nasal polyposis was reported in 35.3% (12/34) of benralizumab patients, contrasting with only 14.3% in both the dupilumab and omalizumab groups; however, this difference was not significant (*p* = 0.34). Rhinosinusitis was present in 11 patients (32.4%) in the benralizumab group and in 2 patients (28.6%) in the omalizumab group, while none were reported in the dupilumab group; the statistical test showed no significant difference (*p* = 0.21). The occurrence of previous surgical procedures for ENT problems was low, as it was reported in four patients (11.8%) in the benralizumab group and in none in the others; *p* = 0.40. Septal deviation was observed in about a quarter of the patients in all groups, with no significant differences (*p* = 0.98) (Table 2).

Pulmonary comorbidities included bronchiectasis, which was more frequent in the dupilumab cohort (71.4%) than in the benralizumab (41.2%) and omalizumab (28.6%) cohorts, but these differences did not reach statistical significance (*p* = 0.23). Pneumonia was very common in all groups, affecting 82.4% of benralizumab patients, 85.7% of dupilumab patients, and 71.4% of omalizumab patients, with no significant variations (*p* = 0.75). No cases of pulmonary nodules or tuberculosis were reported. Sleep-related pulmonary disorders such as OSA were uncommon, with 8.8% in benralizumab and none in the others; their distribution was not statistically significant (*p* = 0.51). Metabolic comorbidities, such as dyslipidemia, were observed in about one-third of patients in all groups (32.4–42.9%), with no significant differences between groups (*p* = 0.49). Obesity affected about 26–28%, with no significant variations (*p* = 0.29). The prevalence of type 2 diabetes was higher in the benralizumab group (29.4%) in comparison with the lack of prevalence in the other groups, approaching significance (*p* = 0.07) (Table 2).

At baseline, all patients, irrespective of the treatment group, had the classic symptoms of severe asthma. Dyspnea, cough, limitation of daily activities, and fatigue were almost universal, affecting 100% of each cohort. Wheezing was reported in 94.1% of the benralizumab patients and in 100% of the dupilumab and omalizumab patients, with no statistically significant differences (*p* = 0.65). Similarly, expectorant cough was present in over 85% of patients in all groups—94.1% in benralizumab, 100% in dupilumab, and 85.7% in omalizumab—with no statistically significant differences (*p* = 0.53). However, nocturnal symptoms were significantly more prevalent in the benralizumab group, with 100% experiencing these symptoms compared with only 57.1% in the omalizumab group (*p* < 0.001).

Symptomatically, all patients experienced hallmark features of severe asthma at baseline, including dyspnea, chronic cough, and activity limitation. Nocturnal symptoms were significantly more prevalent in the benralizumab group (100%) compared with 57.1% in the omalizumab group (*p* < 0.001). Other symptoms, such as wheezing, chest tightness, and nasal congestion, were reported similarly across all groups (Table 3).

Baseline spirometry showed similar lung function in the three groups. The median forced vital capacity (FVC%) was approximately 61.5% for benralizumab, 63.2% for dupilumab, and 62% for omalizumab, with no significant differences (*p* = 0.76). The forced expiratory volume in one second (FEV1%) also appeared comparable, with median values of 48% for benralizumab, 57% for dupilumab, and 69% for omalizumab; this variation was not statistically significant (*p* = 0.24). The inspiratory Tiff index (iTiff), a parameter of airway compression and obstruction, ranged from approximately 60 to 73 within groups (*p* = 0.42). The mean expired airflow, as assessed using the MEF50%, showed lower median percentages in the benralizumab group (18.25%) and higher percentages in the omalizumab group (57%), but the differences were not statistically significant (*p* = 0.16). Overall, these data suggest similar airflow obstruction and baseline lung function at baseline in all groups (Table 4).

At baseline, eosinophil counts were significantly elevated in the benralizumab group, with a median of 750 × 10^3^/μL (460–1035), which was significantly higher than in the dupilumab (median 290) and omalizumab groups (median 270) (*p* = 0.03). IgE levels were significantly elevated in the dupilumab (median 670 IU/mL; 632–831) and omalizumab (median 567 IU/mL; 273–801) groups compared with the benralizumab group (median 128 IU/mL; 75–356), with strong statistical significance (*p* < 0.01). FeNO levels were similar between groups, with medians around 24–45 ppb and no significant differences (*p* = 0.21). The ACT (asthma control test) scores showed a median of 11–14 points, reflecting poor baseline control, with no significant differences between groups (*p* = 0.25) (Table 5).

Following one year of biologic therapy, patients demonstrated substantial improvement across all measured parameters. FVC increased from a median of 62% to 94%, and FEV1 improved from 50% to 81% (*p* < 0.001 for both). MEF50% nearly doubled, and eosinophil counts dropped to undetectable levels. ACT scores improved significantly, from 12 to 25, reflecting better symptom control. The exacerbation frequency decreased from three per year to zero in all groups (Table 6).

Here, an additional file with the figures from the document will be inserted.

The overall results indicated that a substantial proportion of patients achieved significant remission results. Overall, 75% (36/48) achieved biological remission as evidenced by the normalized FeNO levels, and 100% achieved clinical remission, with ACT scores indicating well-controlled symptoms. Improvement in lung function was also notable, with 33.3% (16/48) of patients achieving remission in FEV1 at 6 months, increasing to 75% (36/48) at 12 months. When considering complete remission, which was defined as the concomitant achievement of clinical symptom control, biological control of inflammation (normalization of FeNO), and normalized lung function, 10 patients (20.8%) reached this stage at 6 months, and this number increased to 26 patients (54.2%) at 12 months. This emphasizes that, over time, more and more patients reach a comprehensive state of disease remission, encompassing symptom control, suppression of inflammation, and functional recovery.

In terms of treatment-specific data, 70.6% (24/34) of patients receiving benralizumab (*n* = 34) showed biological remission, with all patients achieving clinical remission and 17.6% (6/34) achieving complete remission at 6 months. At 12 months, biological remission was maintained, and 52.9% of patients (18/34) achieved complete remission. The dupilumab group (*n* = 7) achieved biological remission in 85.7% (6/7), with all patients achieving clinical remission and 42.9% (3/7) achieving complete remission at 6 months, which increased to 71.4% (5/7) at 12 months. The omalizumab group (*n* = 7) achieved biological remission in 85.7% (6/7), and all patients achieved clinical remission, but initial complete remission at 6 months was modest at 14.3% (1/7), increasing to 42.9% (3/7) at 12 months.

Statistical analysis showed no significant differences between treatment groups (*p* > 0.05). These findings highlight that all three biologic drugs can effectively induce a comprehensive remission, covering clinical control, inflammation, and lung function, with progressive improvements over time. The data support the utility of different biological options in achieving long-term disease remission in severe asthma (Table 7).

## 4. Discussions

During treatment, all measured parameters demonstrated significant improvements from baseline (T0) to T3 (final follow-up). Forced vital capacity (FVC%) increased significantly from a median of 62% (interquartile range 52–73%) at T0 to 94% (83–108%) at T3, with a *p*-value of less than 0.001, indicating a substantial improvement in lung volume. Similarly, FEV1% increased from a median of 50% (39–59%) to 81% (72–96%), with an equally significant *p*-value (<0.001). The inspiratory Tiff index (ITiff), a measure of airway collapsibility, showed a median increase from 60 to 68 (*p* < 0.001), reflecting improved airway patency and stability. Small airway function, as assessed by MEF50%, improved from 20% (12–38%) to 46% (32–67%) (*p* < 0.001). Despite the fact that the parameters in the hemogram and other calculated reports were interpreted in our country in batches of eosinophilic versus non-eosinophilic patients [16], our study tried to correlate these biomarkers with obtaining clinical or complete remission.

Although clinical remission was achieved in the entire group of patients, and biological reduction using the FeNo value was achieved in a significant percentage (70%), the inclusion of functional values in the complete remission picture significantly decreased the percentage of patients achieving complete remission. The data are consistent with meta-analyses in the literature [17,18].

Obtaining these results positions the achievement of functional remission according to the literature, which has established a cutoff value of more than 80% or a decrease of no more than 5% from baseline [3,9]. The data from the present study provide a global view of the patient group, regardless of the biologic medication used. A study conducted in 2023 by Dennis Thomas et al. [19] comparatively evaluated two other biologic medications, mepolizumab and omalizumab, and the percentage of patients with clinical remission and lung function criteria was only 23.4% in the omalizumab group. Although the small number of patients included in our study and the different group sizes are limitations of this research, the results are consistent and worth considering. With the increased availability of omalizumab (available in Romania from 2008), the group of patients treated with this product is much lower numerically, on the one hand, due to the more restrictive criteria of inclusion, as well as the prescribing practices. Despite the availability of the first biologic, the reluctance of pneumologists to initiate prescribing will be difficult to overcome in the coming years.

The analysis of risk factors and comorbidities is a strength of the present study, attempting to identify risk factors for suboptimal response to treatment according to the recommendations advocated by Luis Pérez de Llano in 2023 [20]. No direct correlation with incomplete remission was identified. Another limitation of this study is the low number of patients treated with dupilumab, which is related to the timing of its availability on the market in Romania in a compensated regimen (availability in Romania was in the following order: omalizumab, benralizumab, and dupilumab). This situation may explain the low number of ENT comorbidities described in these patients and could explain the suboptimal response.

Inflammatory markers, including the number of eosinophils, decreased significantly from a median of 665 (367–1018) to almost zero (0–30), *p* < 0.001, confirming the strong decrease in eosinophils. FeNO levels decreased from a median of 31 ppb (23–44) to 18 ppb (13–21), indicating reduced airway inflammation (*p* < 0.001). Clinically, ACT scores improved from a median of 12 (11–13) to 25 (24–25), demonstrating significant symptom control (*p* < 0.001). The frequency of exacerbations decreased dramatically from a median of three episodes (2–3) at baseline to zero episodes at follow-up (*p* < 0.001).

Although complete remission is a concept introduced in the treatment of rheumatological diseases [21], the great benefit achieved by rheumatologists is the maintenance of remission even after discontinuation of treatment. For severe asthma, this remains a direction that will have to be concretized in the future prior to remote follow-up [18].

The use of biologic therapies in patients with severe asthma has opened new perspectives on controlling the disease and achieving remission in these patients. The correct use according to national protocols, the choice of the right biologic, and possible switches [22] when needed can increase the likelihood of a favorable outcome and complete remission, even in patients with severe asthma and fixed obstruction [23,24].

The number of patients included in each subgroup (benralizumab, omalizumab, and dupilumab) is not statistically comparable; therefore, a further enlargement of this group and a further development of this study are necessary, as the presented research is a pilot study that opens the way toward the analysis of biologic therapies in Romania.

The results of this study highlight the need for a more personalized approach in the management of severe asthma, which takes into account not only the inflammatory profile but also comorbidities, the age of the onset of the disease, and the therapeutic history [25]. The data obtained indicate that the early initiation of biological therapy, before the development of irreversible airway remodeling, can significantly increase the likelihood of achieving complete remission [23]. In this context, it is necessary to strengthen the continuing medical education of specialists involved in the treatment of severe asthma and promote multidisciplinary collaboration, including that with specialties such as ENT and immunology. At the same time, facilitating access to relevant biomarkers and specialist assessment centers can help to reduce delays in initiating appropriate therapy [26]. These measures can lead not only to an improvement in the clinical prognosis but also to a more efficient use of the resources of the health system.

The cost-effectiveness of biologic therapies in the treatment of severe asthma in Romania is not being assessed by any published studies, despite their importance in substantiating health policy decisions. Despite the suggestion in the international literature that therapies such as omalizumab, benralizumab, and dupilumab may be economical in particular patient subgroups, these findings cannot be directly attributed to the Romanian setting, where cost structure, access to treatment, and population characteristics may differ significantly.

Therefore, it is necessary to develop pharmacoeconomic models adapted to the national context, including parameters such as direct costs, indirect costs (productivity losses), and quality of life indicators (QALYs), in order to accurately assess the impact and sustainability of the use of biologic therapies in Romania.

## 5. Conclusions

Clinical remission is possible in a subset of patients with severe asthma through biologic therapy, as confirmed by this study. These patients demonstrate a positive trend, and there is optimism that they will be able to achieve asthma remission as a realistic therapeutic goal. The results of this pilot study conducted in central Romania validate the data from the literature and align our country with international standards.

## Figures and Tables

**Table 1:** Demographics of patients included in the study.

Demography	Benralizumab (*n* = 34)	Dupilumab (*n* = 7)	Omalizumab (*n* = 7)	*p*
Age (years)	68 (58–73)	65 (55–70)	52 (41–69)	0.08 *
Male-*n* (%)	15 (44.1%)	2 (28.6%)	2 (28.6%)	0.60 **
BMI (kg/m^2^)	27.45 (23.09–33.36)	28.19 (24.15–31.14)	25.71 (23.78–26.60)	0.41 *
Occupational exposure-*n* (%)	16 (47.1%)	2 (28.6%)	1 (14.3%)	0.11 **
Urban-*n* (%)	22 (64.7%)	6 (85.7%)	7 (100%)	0.11 **
Age of asthma onset (years)	50 (37–59)	50 (42–55)	35 (15–42)	0.06 *
Asthma treatment (years)	17 (11–23)	15 (6–17)	15 (4–39)	0.59 *
Smoking-*n* (%)	0 (0%)	0 (0%)	1 (14.3%)	0.06 **
Package/year index	0 (0–11)	8 (0–10)	6 (3–15)	0.28 *
Smoking cessation (years)	0 (0–15)	9 (0–20)	10 (0–13)	0.28 *
Asthma exacerbations in the past year	3 (2–3)	3 (2–3)	2 (2–3)	0.80 *
Asthma-related hospitalizations	1 (1–2)	1 (1–2)	1 (1–2)	0.87 *

* Kruskal–Wallis test; ** Chi-Square test; BMI—body mass index.

**Table 2:** ENT, pulmonary, and metabolic comorbidities.

ENT Comorbidities	Benralizumab (*n* = 34)	Dupilumab (*n* = 7)	Omalizumab (*n* = 7)	*p* **
Allergic rhinitis	10 (29.4%)	2 (28.6%)	2 (28.6%)	0.99
Nasal polyposis	12 (35.3%)	1 (14.3%)	1 (14.3%)	0.34
Rhinosinusitis	11 (32.4%)	0 (0%)	2 (28.6%)	0.21
Previous surgical procedures	4 (11.8%)	0 (0%)	0 (0%)	0.40
Septal deviation	9 (26.5%)	2 (28.6%)	2 (28.6%)	0.98
Pulmonary comorbidities				*p* **
Bronchiectasis	14 (41.2%)	5 (71.4%)	2 (28.6%)	0.23
Pneumonia	28 (82.4%)	6 (85.7%)	5 (71.4%)	0.75
Lung nodules	0 (0%)	0 (0%)	0 (0%)	-
OSAS	3 (8.8%)	0 (0%)	0 (0%)	0.51
Pulmonary tuberculosis	0 (0%)	0 (0%)	0 (0%)	-
Other sleep disorders	0 (0%)	0 (0%)	0 (0%)	-
Metabolic comorbidities				*p* **
Dyslipidemia	11 (32.4%)	3 (42.9%)	1 (14.3%)	0.49
Obesity	9 (26.5%)	2 (28.6%)	0 (0%)	0.29
Type 2 diabetes	10 (29.4%)	0 (0%)	0 (0%)	0.07

** Chi-Square test; ENT—ear, nose, and throat; OSAS—obstructive sleep apnea syndrome.

**Table 3:** Symptoms of patients included in the study.

Symptomatology	Benralizumab (*n* = 34)	Dupilumab (*n* = 7)	Omalizumab (*n* = 7)	*p* **
Dyspnea	34 (100%)	7 (100%)	7 (100%)	-
Cough	34 (100%)	7 (100%)	7 (100%)	-
Limiting daily activities	34 (100%)	7 (100%)	7 (100%)	-
Fatigue	34 (100%)	7 (100%)	7 (100%)	-
Wheezing	32 (94.1%)	7 (100%)	7 (100%)	0.65
Whooping cough	32 (94.1%)	7 (100%)	6 (85.7%)	0.53
Night symptom	34 (100%)	7 (100%)	4 (57.1%)	<0.001
Chest constriction	31 (91.2%)	4 (57.1%)	5 (71.4%)	0.06
Nasal congestion	23 (67.6%)	2 (28.6%)	3 (42.9%)	0.10
Symptoms associated with GERD	0 (0%)	1 (14.3%)	0 (0%)	0.06
Rhinorrhea	24 (70.6%)	3 (42.9%)	3 (42.9%)	0.19
Headache	21 (61.8%)	4 (57.1%)	2 (28.6%)	0.27
Angina	4 (11.8%)	0 (0%)	0 (0%)	0.40

** Chi-Square test; GERD—gastroesophageal reflux disease.

**Table 4:** Initial spirometric values.

Initial Spirometry Values	Benralizumab (*n* = 34)	Dupilumab (*n* = 7)	Omalizumab (*n* = 7)	*p* *
FVC (%)	61.50 (53.15–72.30)	63.20 (51.00–73.90)	62.00 (51.00–100.00)	0.76
FEV1 (%)	48.00 (36.87–58.30)	57.00 (49.00–59.00)	69.00 (39.00–95.00)	0.24
iTiff	60.06 (51.23–68.55)	62.00 (52.30–69.00)	73.17 (57.97–78.00)	0.42
MEF50 (%)	18.25 (12.00–37.45)	24.20 (17.30–32.00)	57.00 (15.00–74.00)	0.16

* Kruskal–Wallis test; FVC—forced vital capacity; FEV1—forced expiratory volume in one second; iTiff—Tiffneau index; MEF50—maximal expiratory flow at 50%.

**Table 5:** Values of biomarkers in the analyzed groups.

Clinical and Paraclinical Reference Parameters	Benralizumab (*n* = 34)	Dupilumab (*n* = 7)	Omalizumab (*n* = 7)	*p* *
Eosinophils (×10^3^/μL)	750 (460–1035)	290 (130–1310)	270 (40–730)	0.03
IgE (U/mL)	128 (75–356)	670 (632–831)	567 (273–801)	<0.01
FeNO (ppb)	34 (25–45)	24 (13–46)	24 (21–40)	0.21
ACT score	12 (11–14)	12 (11–12)	11 (10–12)	0.25

* Kruskal–Wallis test; IgE—immunoglobulin E; FeNO—fractional exhaled nitric oxide; ACT—asthma control test.

**Table 6:** Evolution of the parameters during the study period.

All Patients	T0	T1	T2	T3	*p*
FVC (%)	62 (52–73)	70 (62–92)	84 (71–97)	94 (83–108)	<0.001 ***
FEV1 (%)	50 (39–59)	62 (52–75)	68 (61–85)	81 (72–96)	<0.001 ***
ITiff	60 (51–71)	67 (59–74)	69 (60–57)	68 (63–76)	<0.001 ***
MEF50 (%)	20 (12–38)	31 (23–52)	38 (25–57)	46 (32–67)	<0.001 ***
Eosinophils (×10^3^/μL)	665 (367–1018)	30 (0–160)	0 (0–50)	0 (0–30)	<0.001 ***
ACT score	12 (11–13)	20 (18–22)	22 (21–24)	25 (24–25)	<0.001 ***
Exacerbations	3 (2–3)	0 (0–0)	0 (0–0)	0 (0–0)	<0.001 ***
OCS use	11 (22.9%)	0 (0%)	0 (0%)	0 (0%)	<0.001 **

*** Friedman test; ** Chi-Square test; T0 = baseline, T1 = assessment at 3 months, T2 = assessment at 6 months, T3 = assessment at 12 months; Supplementary File S1. Figures S1–S7. Evolution of FVC, FEV1, Tiffneau index, MEF50, eosinophil counts, and exacerbations over the evaluation timeframes. FVC—forced vital capacity; FEV1—forced expiratory volume in one second; iTiff—Tiffneau index; MEF50—maximal expiratory flow at 50%; ACT—asthma control test; OCS—oral corticosteroid.

**Table 7:** Obtaining clinical/biological remission in the analyzed batch.

	Benralizumab (*n* = 34)	Dupilumab (*n* = 7)	Omalizumab (*n* = 7)	*p* *
Biological remission-FeNO	24 (70.6%)	6 (85.7%)	6 (85.7%)	0.54
ACT clinical remission	34 (100%)	7 (100%)	7 (100%)	-
FEV1 improvement at 6 months	11 (32.4%)	4 (57.1%)	1 (14.3%)	0.23
FEV1 improvement at 12 months	26 (76.5%)	6 (85.7%)	4 (57.1%)	0.43
Complete absence of exacerbations at 12 months	33 (97.1%)	7 (100%)	7 (100%)	0.81
Complete absence of OCS at 12 months	34 (100%)	7 (100%)	7 (100%)	-
Complete remission after 6 months	6 (17.6%)	3 (42.9%)	1 (14.3%)	0.29
Complete remission after 12 months	18 (52.9%)	5 (71.4%)	3 (42.9%)	0.54

* Chi-Square test; FeNO—fractional exhaled nitric oxide; ACT—asthma control test; FEV1—forced expiratory volume in one second; OCS—oral corticosteroid.

## Data Availability

The dataset is available from the authors upon request.

## Supplementary Materials

The following supporting information can be downloaded at: https://www.mdpi.com/article/10.3390/life15071113/s1, Figure S1: Evolution of FVC over the study period; Figure S2: Evolution of FEV1 over the study period; Figure S3: Evolution of Tiffeneau index over the study period; Figure S4: Evolution of MEF50 over the study period; Figure S5: Evolution of eosinophil counts over the study period; Figure S6: Evolution of ACT score over the study period; Figure S7: Evolution of exacerbation counts over the study period.
