# Supplementary material for: Is Biologic Therapy an Effective Tool for Achieving Remission in Severe Asthma? A Retrospective Study in Central Romania
Source: Life (Basel). 2025 Jul 16;15(7):1113. doi: 10.3390/life15071113 (PMC12300431; doi:10.3390/life15071113)
Supplement: Supplementary file 1 [file life-15-01113-s001.zip › life-3719451-supplementary.pdf]

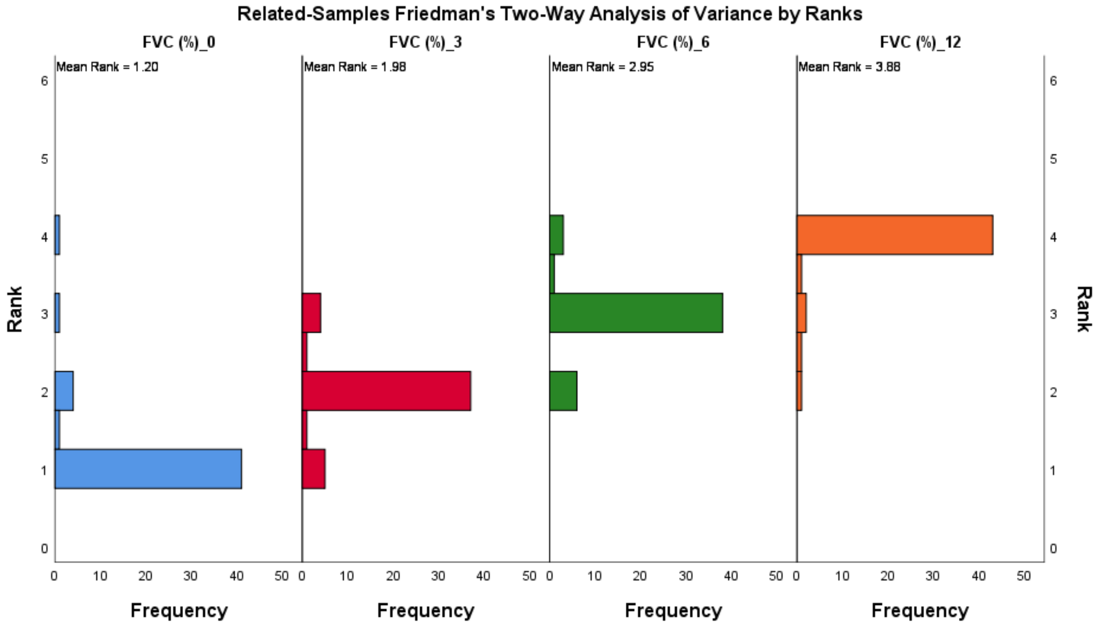

Figure S1: Evolution of FVC over the study period.

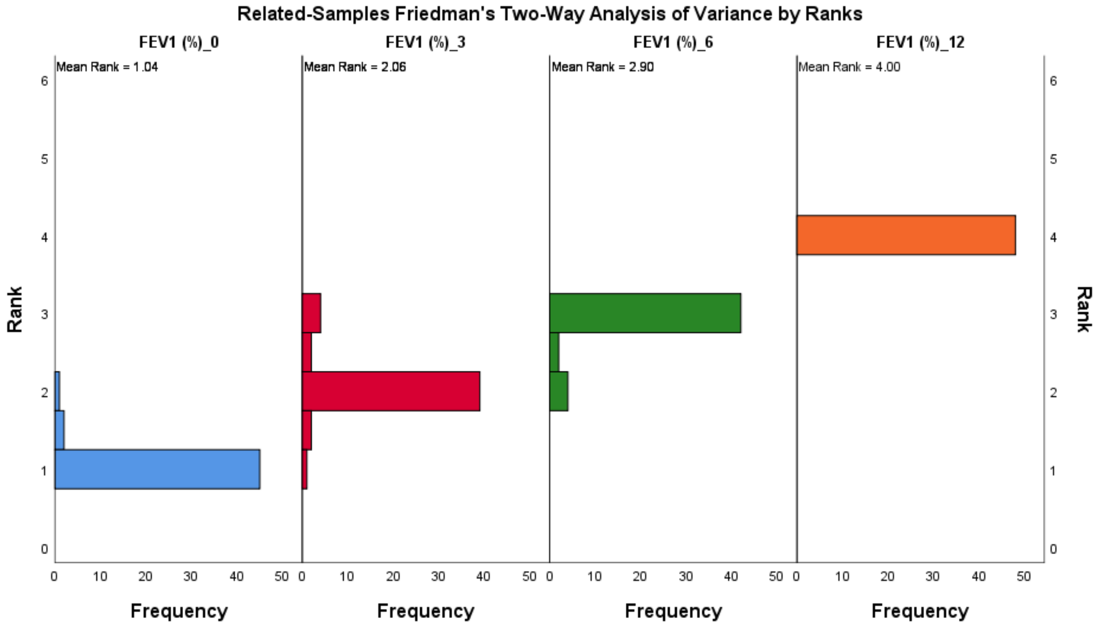

Figure S2: Evolution of FEV1 over the study period.

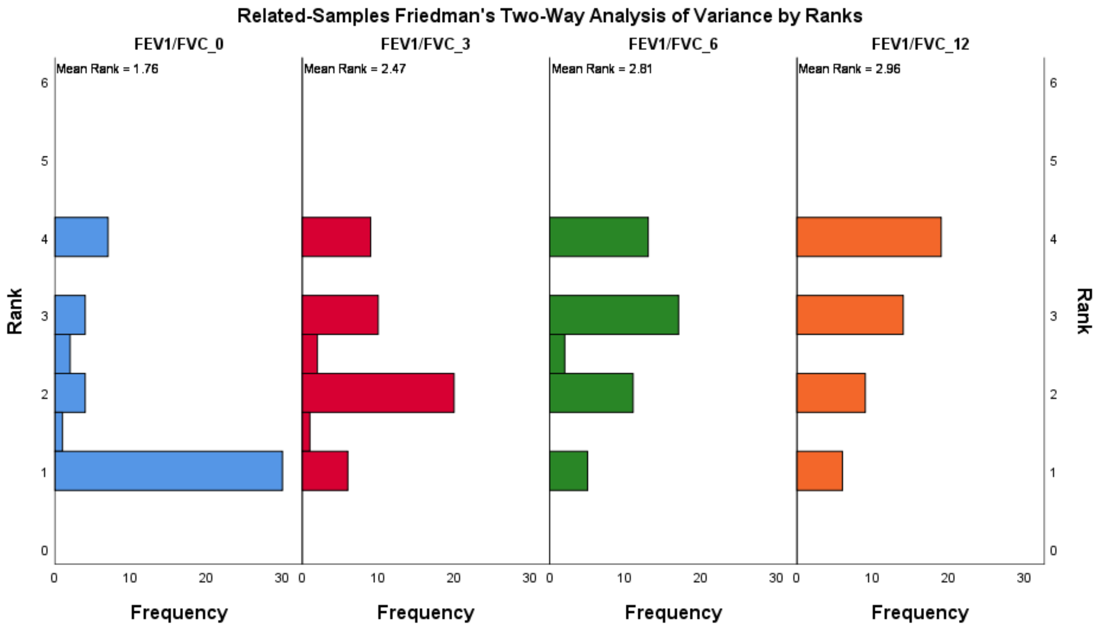

Figure S3: Evolution of Tiffeneau index over the study period.

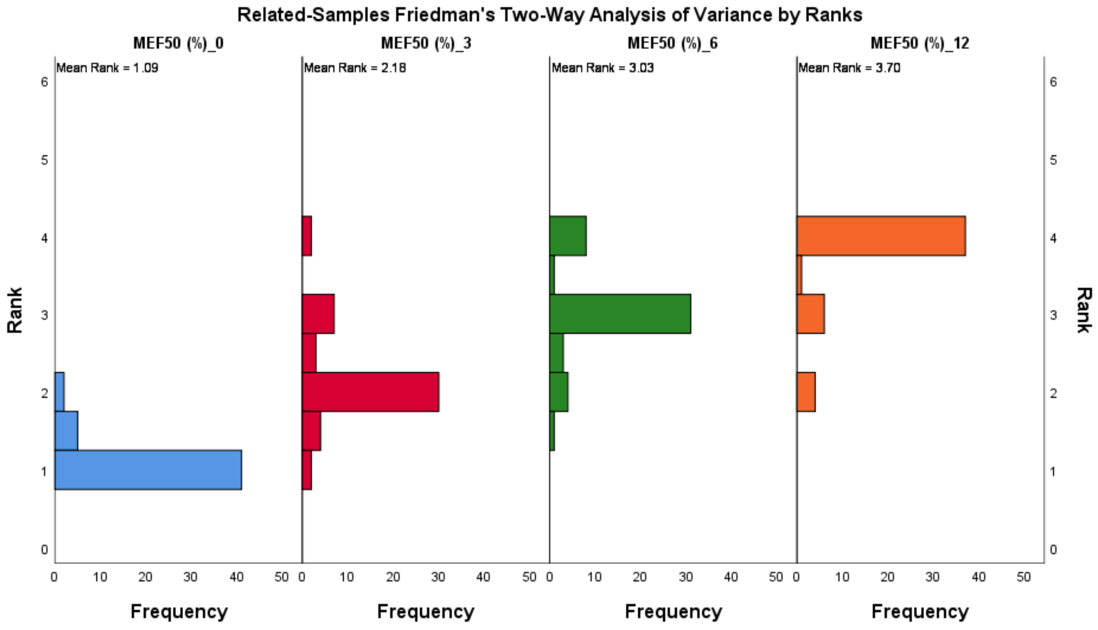

Figure S4: Evolution of MEF50 over the study period.

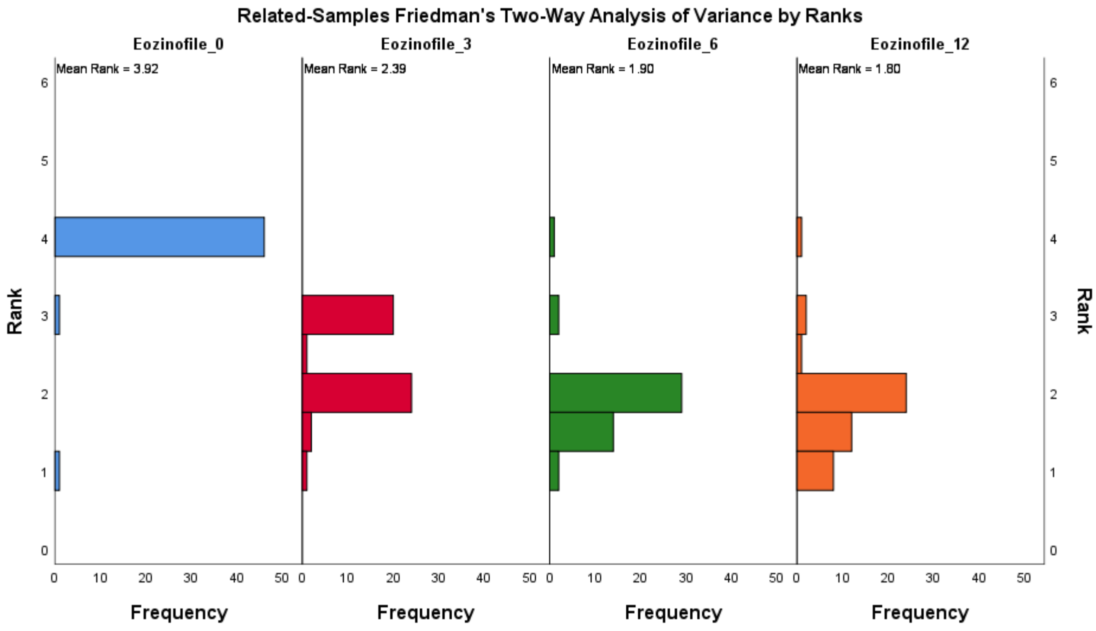

Figure S5: Evolution of eosinophil counts over the study period.

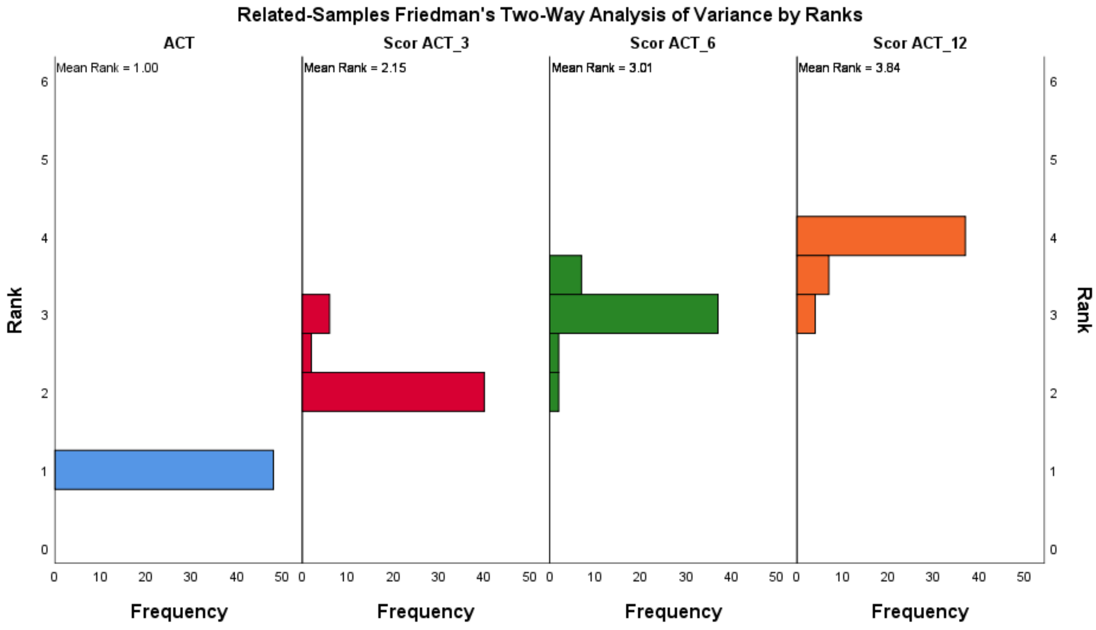

Figure S6: Evolution of ACT score over the study period.

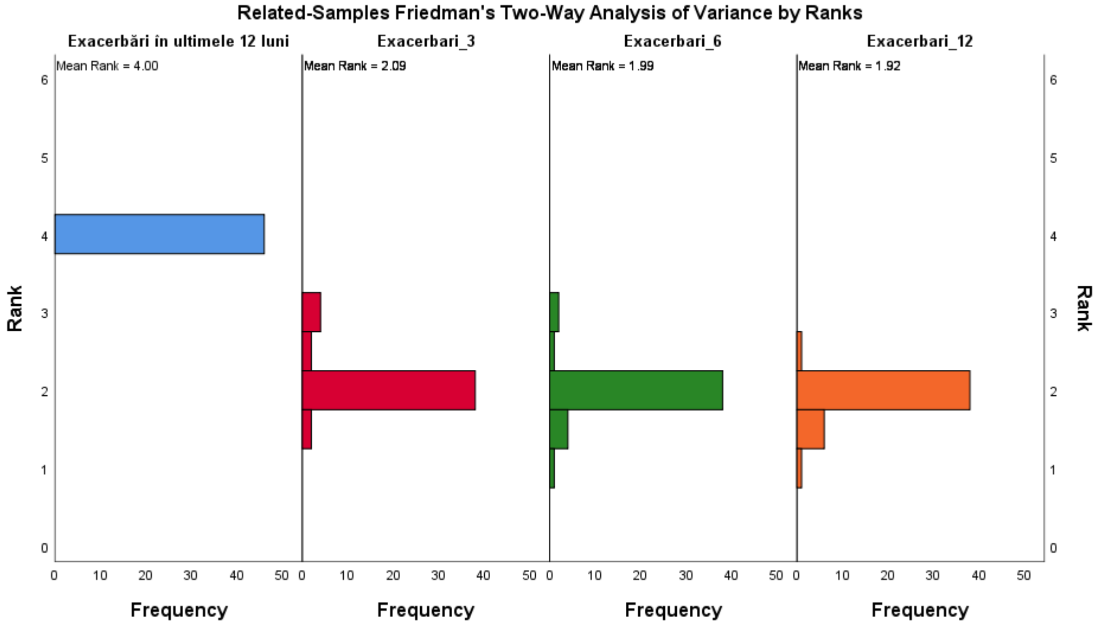

Figure S7: Evolution of exacerbation counts over the study period.
